# Improving mitotic cell counting accuracy and efficiency using phosphohistone‐H3 (PHH3) antibody counterstained with haematoxylin and eosin as part of breast cancer grading

**DOI:** 10.1111/his.14837

**Published:** 2022-11-18

**Authors:** Asmaa Ibrahim, Michael S. Toss, Shorouk Makhlouf, Islam M. Miligy, Fayyaz Minhas, Emad A. Rakha

**Affiliations:** ^1^ Academic Unit for Translational Medical Sciences, School of Medicine University of Nottingham Biodiscovery Institute, University Park Nottingham UK; ^2^ Histopathology department, Faculty of Medicine Suez Canal University Ismailia Egypt; ^3^ Department of Pathology, Faculty of Medicine Assiut University Assiut Egypt; ^4^ Histopathology department, Faculty of Medicine Menoufia University Shebin El Kom Egypt; ^5^ Histopathology department, School of Medicine University of Nottingham Nottingham UK; ^6^ Department of Computer Science University of Warwick Coventry UK

**Keywords:** Breast cancer, count, mitosis, WSI

## Abstract

**Background:**

Mitotic count in breast cancer is an important prognostic marker. Unfortunately, substantial inter‐ and intraobserver variation exists when pathologists manually count mitotic figures. To alleviate this problem, we developed a new technique incorporating both haematoxylin and eosin (H&E) and phosphorylated histone H3 (PHH3), a marker highly specific to mitotic figures, and compared it to visual scoring of mitotic figures using H&E only.

**Methods:**

Two full‐face sections from 97 cases were cut, one stained with H&E only, and the other was stained with PHH3 and counterstained with H&E (PHH3–H&E). Counting mitoses using PHH3–H&E was compared to traditional mitoses scoring using H&E in terms of reproducibility, scoring time, and the ability to detect mitosis hotspots. We assessed the agreement between manual and image analysis‐assisted scoring of mitotic figures using H&E and PHH3–H&E‐stained cells. The diagnostic performance of PHH3 in detecting mitotic figures in terms of sensitivity and specificity was measured. Finally, PHH3 replaced the mitosis score in a multivariate analysis to assess its significance.

**Results:**

Pathologists detected significantly higher mitotic figures using the PHH3–H&E (median ± SD, 20 ± 33) compared with H&E alone (median ± SD, 16 ± 25), *P* < 0.001. The concordance between pathologists in identifying mitotic figures was highest when using the dual PHH3–H&E technique; in addition, it highlighted mitotic figures at low power, allowing better agreement on choosing the hotspot area (*k* = 0.842) in comparison with standard H&E (*k* = 0.625). A better agreement between image analysis‐assisted software and the human eye was observed for PHH3‐stained mitotic figures. When the mitosis score was replaced with PHH3 in a Cox regression model with other grade components, PHH3 was an independent predictor of survival (hazard ratio [HR] 5.66, 95% confidence interval [CI] 1.92–16.69; *P* = 0.002), and even showed a more significant association with breast cancer‐specific survival (BCSS) than mitosis (HR 3.63, 95% CI 1.49–8.86; *P* = 0.005) and Ki67 (*P* = 0.27).

**Conclusion:**

Using PHH3–H&E‐stained slides can reliably be used in routine scoring of mitotic figures and integrating both techniques will compensate for each other's limitations and improve diagnostic accuracy, quality, and precision.

## Graphical abstract

Table 1. Correlation between PHH3 expression with clinicopathological variables; Table 2. Correlation between MAI, with PHH3 and Ki67; Table 3. Multivariate cox regression analysis results for (A) adding PHH3 to predictors of survival (grade and stage), (B) adding PHH3 to mitosis and Ki67 score (C) components of grade (D) components of grade with the replacement of mitosis score with PHH3 score (E) components of grade with the replacement of mitosis score with Ki67 score.

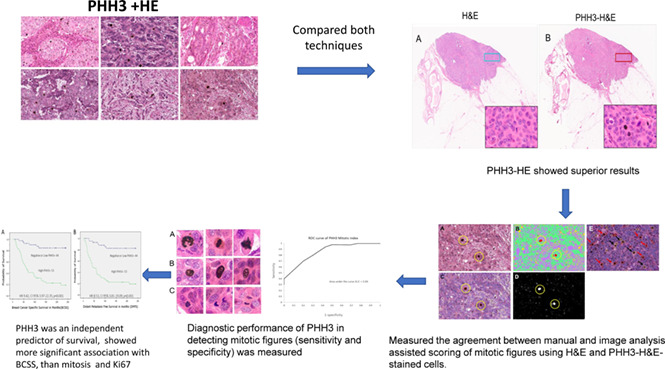

## Background

Mitotic score is a key component of breast cancer (BC) grading and is a strong predictor of survival,^1^ reflecting the underlying biological behaviour of the disease.^2^ However, it is the most time‐consuming component to assess^3^ and is also constrained by low interobserver reproducibility.^4^ Mitotic count discrepancy is considered a frequent cause of overall grade discordance.^5^ The poor reproducibility of mitotic count is mainly attributed to the challenges in detecting mitotically active regions in haematoxylin and eosin (H&E)‐stained slides or the presence of mitotic mimickers such as hyperchromatic nuclei, karyorrhectic or apoptotic cells,^1^, ^6^ even cells in prophase are usually not considered during routine scoring of mitotic figures.^2^ Additionally, the heterogeneity of mitotic activity in different regions, and cell density variations, might all be aggravating factors.^4^, ^7^, ^8^

Histone H3 is one of the five histone proteins that together form the major protein constituents of chromatin in eukaryotic cells.^9^, ^10^ Antibodies directed against phosphorylated histone H3 (PHH3) are almost exclusively expressed in actively proliferating cells during the M phase and late G2 phase,^11^ and are not observed during apoptosis.^12^ The utility of PHH3 has been evaluated in various tumours, including melanoma,^2^, ^13^, ^14^, ^15^, ^16^ neuroendocrine tumours,^2^, ^17^ colorectal and ovarian carcinomas, sarcomas^1^, ^13^, ^16^, ^17^ and central nervous system tumours,^18^, ^19^, ^20^ and revealed correlation with outcome.

Although staining results of both H&E and PHH3 can be viewed using a conventional bright‐field microscope, H&E alone cannot reflect the presence and distribution of underlying specific antigens, just as PHH3 protein expression alone cannot be evaluated adequately without the context of tissue morphology. The dual‐staining technique proposed in this work enables visualization of morphology and molecular profiling over the same tissue section and can thus improve the overall accuracy, quality, and diagnostic precision. Another advantage of this approach is that computational stain separation can be performed on a dual‐stained image to obtain an H&E and an immunohistochemistry (IHC)‐stained whole‐slide image from the same tissue section thus eliminating the need for image registration from serial sections. Consequently, the proposed scheme can be used for the development of computational pathology‐based biomarker prediction algorithms directly from dual‐stained histopathological images without the need for image registration or correspondence analysis.^21^

Combining both H&E and IHC techniques might achieve an optimum method for mitosis detection and counting in BC, and that dual staining of BC tissue sections with PHH3 and H&E will improve the concordance of mitosis counting, and hence the overall grade.

## Materials and methods

This study was conducted on a cohort of primary invasive BC where pseudonymised patient tissue samples were used. Two full‐face tumour sections 4 μm thick from 97 cases were cut; one was stained with H&E only, and the other was stained with PHH3 counterstained with H&E. The cases were selected to represent different grades of BC.

Clinical information and tumour characteristics including patient's age at diagnosis, histological tumour type, grade, tumour size, lymph node status, Nottingham Prognostic Index (NPI), and lymphovascular invasion (LVI) were available. Outcome data were calculated and these included BC‐specific survival (BCSS), defined as the time (in months) from 6 months after the date of primary surgical treatment to the time of death due to BC, and distant metastasis‐free survival (DMFS) defined as the time (in months) from 6 months after surgery until the first event of distant metastasis. Data for oestrogen receptor (ER), progesterone receptor (PR), human epidermal growth factor receptor 2 (HER2), and Ki67 were available as previously published.^22^, ^23^, ^24^, ^25^

ER and PR positivity were defined as positive nuclear staining in ≥1% of the invasive tumour cells.^26^ The proliferation index was evaluated using Ki‐67 antibody staining and defined as high when ≥14% of tumour cells showed nuclear positivity.^27^ Immunoreactivity of HER2 was assessed using Hercep Test guidelines. HER2 positivity was defined as strong positive complete membranous staining in ≥10% of the invasive tumour cells (score 3+). HER2 gene amplification status was assessed in borderline cases (IHC score 2+) using chromogenic *in situ* hybridisation (CISH), using the HER2 CISH pharmDx kit (Dako, Carpinteria, CA, USA), as previously described.^27^, ^28^

### PHH3–H&E counterstaining

Representative paraffin‐embedded tissue blocks of BC tissue were retrieved and processed using a protocol for the dual H&E and IHC staining; 4‐μm tissue sections were cut onto charged slides, and then placed on a 60°C hotplate for 20 min. After rehydration, slides were submerged in citrate buffer at pH 6.0. Water bath heat‐assisted retrieval for 30 min at 96°C was applied with citrate buffer.

**Figure 1:**
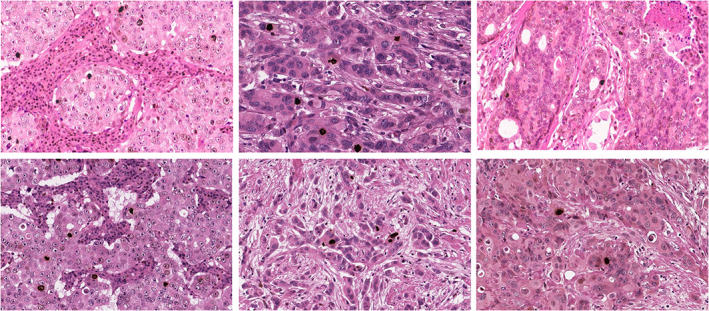
Whole‐slide images (WSIs) were stained with PHH3 and counterstained with H&E at 40× magnification.

Rabbit polyclonal anti PHH3 (Abcam, Cambridge, MA, USA; phospho S10 antibody, ab5176) was diluted at 1:100 in Leica antibody diluent (RE AR9352, Leica, Biosystems, Newcastle upon Tyne, UK) and incubated with the sections for 60 min at room temperature. The DAB (Novolink kit, Leica, Biosystems) working solution was applied. Haematoxylin nuclear stain was applied for a longer period (8 min), to remove nonspecific background staining and to improve contrast, weak acid alcohol was used, and then eosin counterstain was applied (2 min); Figure 1. Tonsil tissue was used as a positive control.

Stained slides were scanned at 40× magnification using a high‐throughput slide scanner (Pannoramic 250 Flash III; 3DHistech, Budapest, Hungary), and the slides were then viewed with case viewer software program (v. 2.2.0.85; 3D‐Histech).

### Mitotic counts on H&E slides and PHH3–H&E dual‐stained sections

We assessed the utility of adding PHH3 to routine H&E in scoring mitosis and grading BC by comparing counting mitosis using this technique with traditional mitoses scoring using H&E only.

### Interobserver agreement in detecting mitotic figures

**Figure 2:**
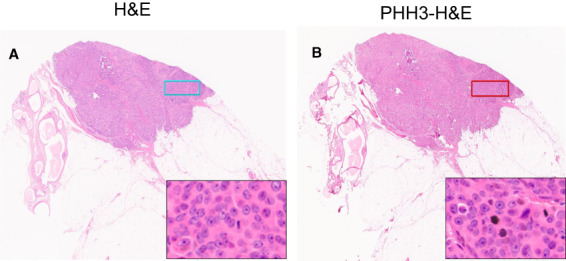
**A**: WSI stained with H&E only at 0.5× magnification. **B**: WSI stained with PHH3 and counterstained with H&E at 0.5× magnification. A 3 mm^2^ rectangle, drawn in the same region in each slide using a grid; inset images show different staining techniques.

For assessment of the reproducibility of each staining technique, two sections from each case were utilised, one stained with H&E only and the other was stained with PHH3 and counterstained with H&E. A 3 mm^2^ rectangle was drawn, in the exact region in each of the two slides, and mitotic figures within each rectangle were counted: Figure 2.

Mitotic counts using H&E and dual PHH3–H&E immunostaining techniques were independently scored by two certified pathologists to measure the agreement between them.

The technique that achieved the highest level of agreement was considered the most reliable one. For each staining technique, the average time required to count mitoses was recorded.

### Interobserver concordance on hotspot identification

To determine the most effective method for revealing the greatest number of mitotic figures (hotspots), we evaluated the agreement of two pathologists in detecting mitotic hotspots in 20 whole‐slide images (WSIs) by having each of them draw a 5‐mm^2^ circle in the area with the highest number of mitotic figures using the circle annotation tool in the toolbar. Agreement was reached when these circles overlapped or intersected.

### Image analysis‐assisted PHH3 indices

We assessed the degree of agreement between manual and digital image analysis (DIA) tools (ImageJ, NIH, Bethesda, MD, USA [v1.53f51]^29^ and QuPath [v0.3.1; Queen's University Belfast, Belfast, UK]^30^) in counting mitoses using PHH3–H&E and conventional H&E‐stained slides, in addition to quantifying the number of PHH3‐stained G2 phase‐stained cells using 40 images at 40× magnification.

### Measurement of accuracy (sensitivity and specificity) of PHH3–H&E IHC staining

**Figure 3:**
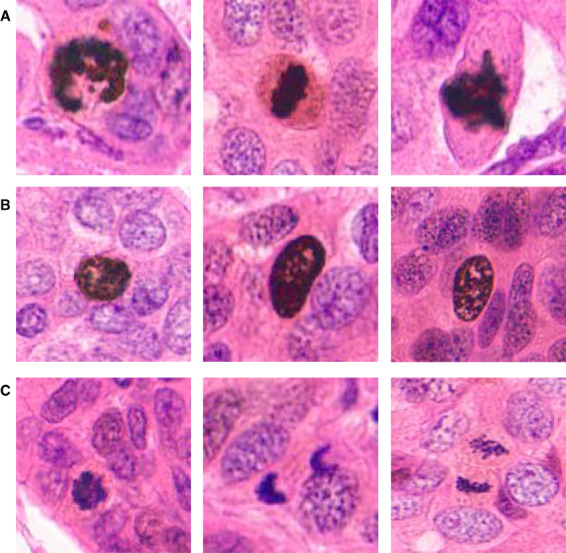
**A**: Showing true positive mitotic figures. **B**: Showing PHH3‐stained cells in the G2 phase of the cell cycle. **C**: Shows some of the few missed (false‐negative) mitotic figures. [Color figure can be viewed at wileyonlinelibrary.com]

Using this method, we were able to assess PHH3's diagnostic performance and accuracy in detecting true mitotic figures. The relative ability of PHH3 to distinguish mitotic figures from other cells in the cell cycle was determined by performing the receiver operating characteristic (ROC) curve. ROC curves demonstrate the coordinate variation in sensitivity (shown on the *Y*‐axis) and specificity (shown on the *X*‐axis) of a test as the threshold for defining test positivity, which varies over the entire range of possible test outcomes. Sensitivity and specificity were calculated as follows:

- Brown‐stained nuclei with loss of nuclear membrane or the presence of chromosome condensation arranged along a plane or separated were considered *true‐positive mitotic figures*.
- Unstained or missed mitotic figures showing the above criteria were considered *false‐negative mitotic figures*.
- While intact brown‐stained nuclei or nuclei with smooth membrane and the absence of chromosome condensation were considered *false‐positive mitotic figures*, or *PHH3‐stained G2 phase cells* ^8^; Figure 3.

### Reassessment of the mitotic score and histological grade based on the mitotic activity index (MAI) versus PHH3

The number of mitotic figures stained by PHH3–H&E was compared with those stained with H&E only, both counted in each slide within the same 3 mm^2^ areas of highest mitotic activity. The counted mitotic number was converted to a score according to the Nottingham grading system, as follows: mitosis score 1 for less than or equal to 11 mitoses per 3 mm^2^, mitosis score 2 from 12–22 mitoses per 3 mm^2^; mitosis score 3 for equal to or greater than 23 mitoses 3 mm^2^. These newly scored PHH3‐stained mitotic figures were compared to the mitosis score assessed by the MAI of H&E slides.

### Statistical analysis

All statistical analyses were performed using SPSS v. 26 (IBM, Armonk, NY, USA). The correlations between categorical variables were analysed by the Chi‐square test. The total number of PHH3‐stained mitotic figures was dichotomised based on BCSS using X‐tile bioinformatics software version 3.6.1 (School of Medicine, Yale University, New Haven, CT, USA)^31^ into high (≥20 mitoses/3 mm^2^) and low (<20 mitoses /3 mm^2^). Differences between the two independent groups were compared by the Mann–Whitney *U*‐test. The degree of interobserver agreement was assessed using the intraclass correlation coefficient (ICC) for continuous data. The Kappa statistic was used to assess the concordance between observers for categorical variables. Outcome analysis was assessed using Kaplan–Meier curves and the log‐rank test. The Cox regression model was used for the univariate and multivariate analysis. For all tests, *P* < 0.05 (two‐tailed) was considered statistically significant.

## Results

### Performance of using PHH3–H&E mitotic count in comparison with H&E‐stained mitotic figures

**Figure 4:**
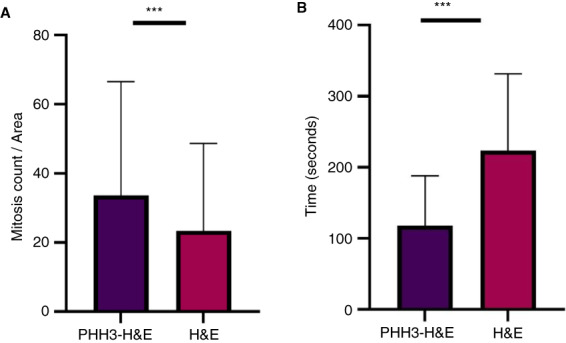
**A**: Mitotic count between H&E and PHH3–H&E technique (*P* < 0.001). **B**: Average time consumption for pathologists using H&E only and PHH3–H&E technique (*P* < 0.001). [Color figure can be viewed at wileyonlinelibrary.com]

When using H&E stain only, the number of mitoses was significantly underestimated as compared to those identified using the PHH3–H&E staining technique; Figure 4A.

Pathologists detected significantly higher mitotic figures using the PHH3–H&E (median ± SD, 20 ± 33) compared with the H&E method (median ± SD, 16 ± 25), *P* < 0.001.

### Interobserver variability in detecting mitosis on H&E and with PHH3– H&E

High agreement between pathologists was observed when using PHH3–H&E (ICC = 0.820) in comparison with standard H&E (ICC = 0.514). The concordance between pathologists in identifying mitotic figures was highest when using the dual PHH3–H&E technique and was lowest using H&E‐stained slides only.

For both pathologists, the time taken to score mitotic figures stained with H&E only was significantly longer than the scoring time for those mitotic figures stained with PHH3–H&E (median ± SD, 240 ± 108 sec/3 mm^2^ for HE only and median ± SD, 120 ± 70 sec/3 mm^2^ for PHH3–H&E; *P* < 0.001); Figure 4B.

### Interobserver concordance on hotspot identification

PHH3‐labelled‐mitotic figures were easily seen and permitted quick identification of hotspots; it highlighted mitotic figures at low power at ease without strain. Agreement between pathologists when using PHH3–H&E (*k* = 0.842) was better in comparison with H&E (*k* = 0.605).

### Image analysis assisted PHH3 indices

Counting H&E as well as PHH3‐stained mitotic cells was performed using ImageJ and QuPath software and compared with an experienced pathologist eye using digitalised WSIs.

For H&E‐stained mitotic figures, a fair agreement between QuPath and ImageJ (ICC = 0.431), between ImageJ and pathologist eye (ICC = 0.337), and between pathologist and QuPath (ICC = 0.405) was observed.

**Figure 5:**
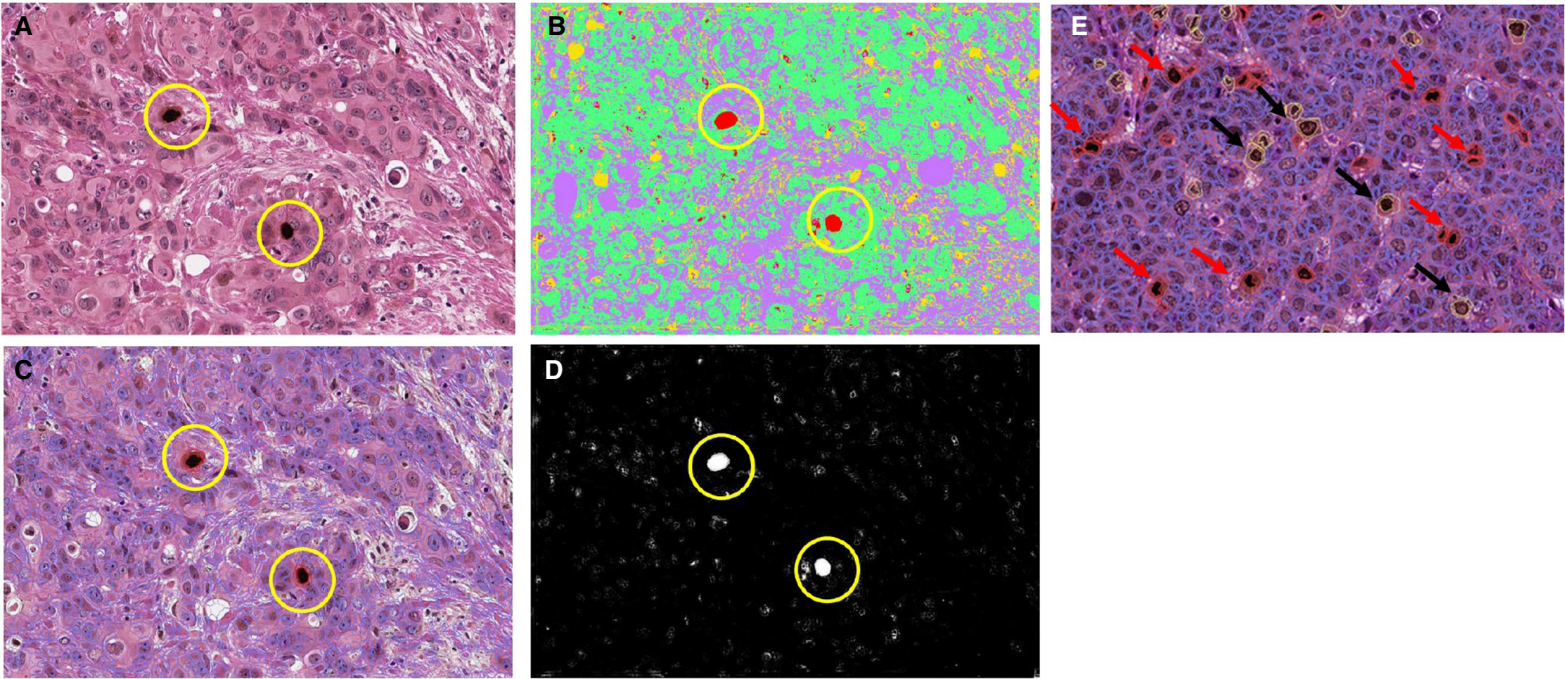
**A**: Whole‐slide image (WSI) stained with PHH3 and counterstained with H&E at 40× magnification, showing two identified mitotic figures (yellow circle). **B**: The same image using QuPath software; it identified the same mitotic figures (yellow circles). **C,D**: The same image using ImageJ software; it identified the same mitotic figures (yellow circles). **E**: QuPath software showing distinction between PHH3‐stained mitotic figures (red arrows) and PHH3‐stained G2 cells (black arrows). [Color figure can be viewed at wileyonlinelibrary.com]

For PHH3‐stained mitotic figures, a good agreement between QuPath and ImageJ was observed (ICC = 0.692), between ImageJ and pathologist eye (ICC = 0.706), and between pathologist and QuPath (ICC = 0.824); Figure 5.

Regarding the distinction between PHH3‐stained mitotic cells and G2 cells, a good agreement was observed between QuPath and ImageJ (ICC = 0.643), ImageJ and pathologist eye (ICC = 0.791), and between pathologist and QuPath (ICC = 0.834), in detecting PHH3‐stained G2 cells only.

### Sensitivity and specificity of PHH3–H&E immunohistochemistry staining in counting mitotic figures

**Figure 6:**
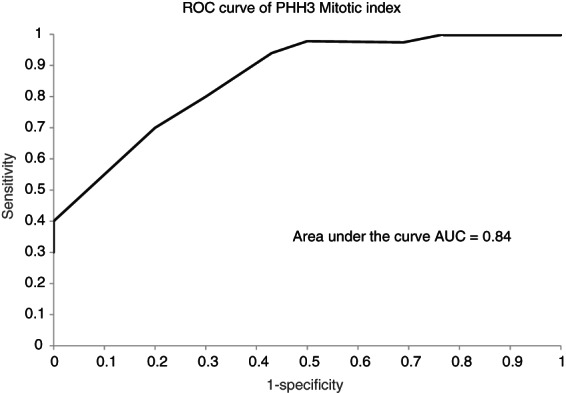
ROC curves represent the diagnostic accuracy of PHH3, in detecting true mitotic figures.

PHH3 diagnostic performance using diagnostic testing metrics such as the sensitivity, specificity, and area under the ROC curve (AUC), revealed that AUC was equal to 0.84, suggesting that PHH3 can be used as a good accurate test in detecting mitotic figures; Figure 6.

### Reassessment of the mitotic score and histological grade based on the MAI versus PHH3

Using PHH3–H&E, 9 cases of grade 1 were upgraded to grade 2 and 15 cases of grade 2 were upgraded to grade 3 (a total of 24 upgraded cases). None of the cases were downgraded.

### Associations between PHH3 expression and clinicopathological parameters of BC

The associations between the PHH3 expression level and clinicopathological features of the tumours are summarised in Table 1.

**Table 1:** Correlation between PHH3 expression with clinicopathological variables

Parameter	PHH3 expression		
	Low No (%)	High No (%)	*x* ^2^ *P‐*value
Patient age (years)			
<50	12 (40)	18 (60)	0.504
≥50	32 (47.8)	35 (52.2)	0.478
Tumour size (cm)			
<2 cm	37 (75.5)	12 (24.5)	36.316
≥2 cm	7 (14.6)	41 (85.4)	**<0.001**
Tumour grade			
1	26 (96.3)	1 (3.7)	50.803
2	8 (72.7)	3 (27.3)	**<0.001**
3	10 (17)	49 (83)	
Tubule formation			
1	22 (100)	0 (0)	36.546
2	7 (46.7)	8 (53.3)	**<0.001**
3	15 (25)	45 (75)	
Mitotic score			
1	33 (97.1)	1 (2.9)	57.936
2	3 (37.5)	5 (62.5)	**<0.001**
3	8 (14.5)	47 (85.5)	
Nuclear pleomorphism			
1	21 (100)	0 (0)	39.904
2	8 (66.7)	4 (33.3)	**<0.001**
3	15 (23.4)	49 (76.6)	
Molecular subtypes			
Luminal A	21 (91.3)	2 (8.7)	
Luminal B	5 (18.5)	22 (81.5)	43.04
HER2	0 (0)	5 (100)	**<0.001**
TNBC	4 (18.2)	18 (81.8)	
Histological subtypes			
Nonspecific type (NST)	14 (49.2)	7 (50.8)	40.442
Lobular	22 (100)	0 (0)	**<0.001**
Mixed NST and special type	1 (100)	0 (0)	
Other special types*	7 (53.8)	6 (46.2)	
Axillary nodal stage			
Stage 1	36 (56.3)	28 (43.8)	9.245
Stage 2	6 (27.3)	16 (72.7)	**0.01**
Stage 3	2 (18.2)	9 (81.8)	
Nottingham Prognostic Index			
Good	32 (97)	1 (3)	53.753
Moderate	7 (18.4)	31 (81.6)	
Poor	5 (19.2)	21 (80.8)	**<0.001**
Lympho‐vascular invasion			
Negative	37 (57.8)	27 (42.2)	11.768
Positive	7 (21.2)	26 (78.8)	**0.001**
Oestrogen receptor			
Negative	6 (18.2)	27 (81.8)	16.282
Positive	38 (58.1)	26 (41.9)	**<0.001**
Progesterone receptor			
Negative	14 (30.4)	32 (69.6)	8.254
Positive	22 (56.4)	17 (43.6)	**0.016**
Missing	8 (66.7)	4 (33.3)	
Human epidermal growth factor receptor 2 (HER2) status			
Negative	34 (43.6)	44 (56.4)	4.867
Positive	3 (30)	7 (70)	0.088
Triple negative status			
Nontriple negative	36 (53.7)	31 (46.3)	8.521
Triple negative	4 (18.2)	18 (81.8)	**0.014**
Ki67 index			
Low	25 (86.2)	4 (13.8)	51.588
High	7 (13)	47 (87)	**<0.001**
Missing	12 (85.7)	2 (14.3)	

* *P*‐value in bold is significant.

PHH3‐positivity was significantly associated with aggressive characteristics, including higher tumour stage (*P* = 0.01), tumour size ≥2 cm, high grade, nuclear pleomorphism, few tubule formations, and poor NPI (*P* < 0.001).

### Correlation of PHH3 with MAI and Ki67

A strong positive significant correlation was found between mitotic count per 3 mm^2^ and PHH3 score (*r* = 0.738, *P* < 0.001), while a weak positive correlation was observed for the Ki67 score (*r* = 0.269, *P* = 0.01); Table 2. A weak positive correlation was found between PHH3 and Ki67 (*r* = 0.177, *P* = 0.016).

**Table 2:** Correlation between MAI, with PHH3 and Ki67

Pearson correlation coefficient	Ki67 index groups	PHH3 score
Mitotic activity index (MAI)	0.269**	0.738**
*P*‐value	0.01	<0.001

** Statistically significant.

### Outcome analysis

**Figure 7:**
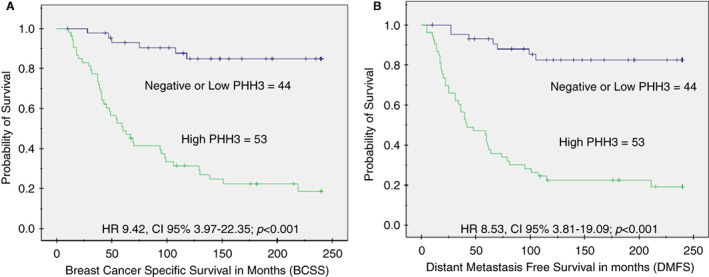
Kaplan–Meier survival curves show the association between high PHH3 expression and poor outcome in terms of **(A)** BCSS and **(B)** DMFS. [Color figure can be viewed at wileyonlinelibrary.com]

Univariate survival analysis revealed that patients with a high number of PHH3‐stained mitotic figures with a cutoff of PHH3 mitotic figures >20 per 3 mm^2^, had a significantly shorter BCSS and DMFS (hazard ratio [HR] 9.42, 95% confidence interval [CI] 3.97–22.35; *P* < 0.001) and (HR 8.53, 95% CI 3.81–19.09; *P* < 0.001) respectively; Figure 7.

In the nonchemotherapy‐treated cohort, a high number of PHH3‐stained mitotic cells were predictive of a higher risk of death from BC (*P* < 0.001), and occurrence of distant metastasis (*P* < 0.001). However, such an association was not observed in patients who received chemotherapy.

**Figure 8:**
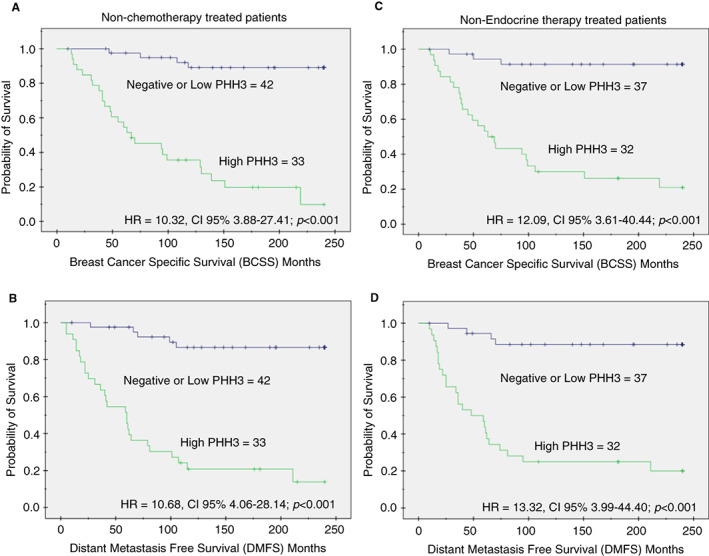
Kaplan–Meier survival curves showing the association between high PHH3 expression and poor outcome in terms of **(A)** BCSS and **(B)** DMFS, in nonchemotherapy and **(C)** BCSS and **(D)** DMFS nonhormonal therapy treated cohorts. [Color figure can be viewed at wileyonlinelibrary.com]

Similarly, in the nonhormonal therapy‐treated cohort, high PHH3 was predictive of a higher risk of death from BC (*P* < 0.001), and occurrence of distant metastasis (*P* < 0.001). However, such an association was not observed in patients who received hormonal therapy; Figure 8.

In the multivariate Cox regression model including other prognostic covariates (tumour grade and nodal stage), PHH3 was an independent predictor of shorter BCSS (HR 2.568, 95% CI 1.05–6.28; *P* = 0.039) and worse DMFS (HR 2.87, 95% CI 1.24–6.67; *P* = 0.014). When PHH3 was added to mitosis and the Ki 67 score, it was an independent predictor of BCSS (HR 5.94, 95% CI 2.37–14.89; *P* < 0.001), and DMFS (HR 4.63, 95% CI 2.13–10.04; *P* < 0.001), and when mitosis was replaced with PHH3 in a Cox regression model with other grade components, PHH3 was an independent predictor of survival (HR 5.66, 95% CI 1.92–16.69; *P* = 0.002), and even showed more significant association with BCSS than mitosis (HR 3.63, 95% CI 1.49–8.86; *P* = 0.005) and Ki67 (*P* = 0.27) in our sample; Table 3.

**Table 3:** Multivariate Cox regression analysis results for (a) adding PHH3 to predictors of survival (grade and stage), (B) adding PHH3 to mitosis and Ki67 score, (C) components of grade (D) components of grade with the replacement of mitosis score with PHH3 score (E) components of grade with the replacement of mitosis score with Ki67 score

		BCSS				DMFS			
		Hazard ratio (HR)	95% confidence interval (CI)		*P* value	Hazard ratio (HR)	95% confidence interval (CI)		*P* value
			Lower	Upper			Lower	Upper	
**A**	***PHH3 Score***	2.568	1.050	6.282	**0.039**	2.873	1.237	6.673	**0.014**
	***Grade***	5.194	1.825	14.779	**0.002**	3.557	1.573	8.046	**0.002**
	***Stage***	1.524	1.017	2.284	**0.041**	1.723	1.149	2.582	**0.008**
**B**	***Mitosis Score***	2.044	0.813	5.136	**0.013**	2.305	0.956	5.558	0.063
	***PHH3 Score***	5.943	2.372	14.891	**<0.001**	4.629	2.134	10.041	**<0.001**
	***Ki67 Score***	1.001	0.999	1.002	0.284	1.001	1.000	1.002	0.136
**C**	***Tubular formation***	1.572	0.597	4.138	0.360	1.163	0.492	2.747	0.731
	***Pleomorphism***	3.114	0.865	11.208	0.082	2.692	0.924	7.841	0.069
	***Mitosis Score***	3.635	1.492	8.858	**0.005**	3.956	1.684	9.294	**0.002**
**D**	***Tubular formation***	2.383	0.868	6.544	0.092	1.743	0.714	4.257	0.222
	***Pleomorphism***	0.707	0.145	3.445	0.667	0.672	0.171	2.638	0.568
	***PHH3 Score***	5.660	1.918	16.699	**0.002**	5.118	1.956	13.391	**0.001**
**E**	***Tubular formation***	1.766	0.657	4.752	0.260	1.313	0.540	3.193	0.549
	***Pleomorphism***	5.075	1.342	19.199	**0.017**	4.610	1.486	14.304	**0.008**
	***Ki67 Score***	1.001	0.999	1.002	0.276	1.001	1.000	1.002	0.165

***P*** value in bold is significant.

## Discussion

In the UK, it is estimated that over 13 million histopathological cases are examined annually, averaging 65,000 slides per day; the majority of these cases require scoring of mitosis as part of the assessment of the proliferative capacity and for prognostic classification.^31^

Nearly 55,920 cases are diagnosed with BC each year,^32^ and accurate assessment of mitotic activity in these cases is essential for tumour grading and in predicting the risk of disease progression. BCs are graded based on mitotic count into scores 1, 2, and 3 on standard H&E‐stained slides, which is a relatively subjective and time‐consuming task.^33^, ^34^

There are many approaches for assessing the proliferation (growth) potential of tumours, including assessing the overall proliferation index (average mitotic score), and mitotic evaluation in randomly selected areas and the highly mitotic areas of the tumour (hotspots). In a previous study carried out by our group, we found that there is a tendency to underestimate mitotic count in randomly selected areas or the whole tumour slide compared to hotspots.^35^ The mitotic activity is used to reflect tumour cell division and growth potential. Therefore, the highest mitotically active tumours areas are important to be identified, as these are the most likely to progress and respond to cytotoxic chemotherapeutic agents. Other comparative studies between the methods of assessment of mitotic counts or the proliferation activity of breast cancer showed that evaluation in the highly proliferative pool of the tumour (hotspots) is the best representative indicator for the behaviour of the tumour and is strongly associated with the outcome.^36^ In line with this, the current breast cancer guidelines recommend counting mitoses within the hotspots to define the proliferation score and grade of breast cancer.^37^, ^38^

Accurate histologic grading is required for effective clinical staging and treatment decisions; however, distinguishing mitotic figures in H&E‐stained slides from similar chromatin changes is a subjective process that can be subjected to intra‐ and interobserver variation.^39^ PHH3 has the benefit of being relatively mitosis‐specific, detecting cells during their transition from the G2 to M phase.^40^

Our study evaluated this subjectivity by assessing the interobserver reproducibility of mitotic count using this new technique of counterstaining PHH3 with H&E among pathologists, we have found that the agreement among pathologists in recognizing mitotic figures was highest when employing the dual PHH3–H&E staining approach. In accordance with other studies,^41^, ^42^ we have also found that the number of mitoses was dramatically undercounted when H&E stain was used alone as opposed to the PHH3–H&E staining method.

Moreover, PHH3 staining within a given tumour was heterogenous and allowed for easy identification of mitotic hotspots; lastly, it was significantly less time‐consuming than counting mitoses on conventional H&E preparations, sparing precious diagnostic time, and efficiently increasing the number of cases diagnosed daily, while improving the quality of diagnosis.

The added value of using PHH3–H&E immunostaining is that it allows pathologists to assess the morphologic features of mitosis at the same time, with the tumour histological features increasing the specificity of quantification.^8^

We also consider that using H&E in staining along with PHH3, or other diagnostic antibodies, can spare important diagnostic areas that could be lost during sequential sectioning, sparing the valuable tissue biopsies as serial sectioning may cut through the area of interest and may result in the loss of regions necessary for critical diagnosis. This is particularly an issue with smaller core needle biopsies that are of limited size and number. And if we considered the removal of the H&E stains, it does not always leave the target epitopes intact for potential reuse of the slide for selective biomarkers in current existing protocols.^43^ For this reason, an innovative method utilizing IHC–H&E on the same slide without destaining can spare the tissue without sequential cutting.

Another advantage of using dual‐stained slides is that the rapidly expanding use of WSI and artificial intelligence allowed the use of more objective measurements, including DIA for more accurate and objective grade reporting.^44^ And using coloured indices such as DAB would be much easier for identification and quantification than the morphological subjective criteria.

Thus, mitotic count based on PHH3 staining appears a robust, easy, and reliable method and could potentially decrease interobserver variability, especially with less experienced pathologists.

We also demonstrated that using ImageJ analysis‐assisted techniques was comparable to the human eye in terms of the detection of mitotic figures, and the agreement even improved when these mitotic figures were labelled with PHH3, and the distinction between PHH3‐labelled mitotic figures and G2 phase‐stained cells are possible with good agreement.

Using this technique, we were able to test the accuracy of mitosis detection by PHH3, and it showed high accuracy reflected by the sensitivity, specificity, and ROC curve. Despite missing a few mitotic cells, this may be due to IHC‐related technical issues with tissue fixation and antigenic retrieval.

We examined the clinical outcome of the patients, and based on our findings we found that PHH3 has the capability for a further contribution to BC grading and classification, and could be especially beneficial for pathologists, and training machine‐learning algorithms.

The mitotic count showed a significant positive association with PHH3 score, per 3 mm^2^, whereas the Ki67 score showed only a mild positive correlation. Although Ki67 is a widely used and well‐known proliferation marker in BC, it is not specific for mitosis, but is expressed in all phases of the cell cycle. Many cells that are not committed to cell division (not in the mitosis phase of the cell cycle) will be positive for Ki67. In contrast, PHH3 specifically identifies cells undergoing mitosis; therefore, it would provide a better representation of proliferation activity in BC and can be used in the clinical setting to identify mitoses.

PHH3 was an independent predictor of survival when it was added to grade and nodal stage, and it even showed a more significant association with survival than mitosis score, and Ki67 in the multivariate analysis; therefore, the PHH3 score could be more predictive of outcome than mitosis and Ki67. This agrees with other studies, where it has been proposed as a replacement for the Ki67 index in several cancers.^45^, ^46^, ^47^ A higher significant association with the patient outcome with a higher hazard ratio was associated with the PHH3 score than the mitosis scores, which supports the hypothesis that PHH3 could replace the mitosis score in grading and could improve BC behaviour prediction and the grading scheme.

A challenge that might face the implementation of PHH3 staining in routine practice is the cost burden on the pathology service, especially in places where healthcare is not extensively subsidised. It would be a trade‐off between precision and expense. Healthcare providers in general and pathologists specifically should supply the best possible service to the patients whenever possible and they should be responsible for the decision and diagnoses made. Another point to mention is that PHH3 staining has the same cost as other routinely assessed IHC markers in BC such as ER and Her2, and can provide prognostic value at a lower cost than existing multigene assays and will refine BC grading when using WSIs, which are associated with lower mitoses detection ability^48^; it has been shown to be more time‐consuming than counting using conventional microscopes.^49^ The selective approach could be a solution where targeted patients can benefit more from PHH3 staining and assessment, especially poorly fixed specimens or in borderline cases between mitosis scores 1 and 2 or 2 and 3, where such scores may affect the overall BC grading and hence patient management. In these instances, it would alleviate cost concerns.

Moreover, utilizing PHH3 to refine mitosis counting, requires readjusting the range and the cutoffs used to define mitosis scores in BC, as it was shown that the number of mitotic figures detected using PHH3 is higher than that detected using H&E. This refining would need multicentric validation on a large number of cases and with long follow ‐p data.

## Conclusion

Histopathological diagnoses of tumours depend mainly on H&E and IHC staining. These are the gold standards in clinical care. We are developing a new technique that combines both and can be tissue‐ and timesaving, while improving the diagnostic quality. It provides a more sensitive approach for training artificial intelligence IHC prediction models while using the exact same slide. Our results demonstrated a tendency to undergrade BCs based on H&E compared with PHH3, which alters the stage, risk of disease progression, and treatment recommendations. We, therefore, show for the first time the potential of using PHH3 counterstained with H&E for precise routine mitotic scoring in clinical practice.

### Statement of Ethics

This work was approved by the Nottingham Research Ethics Committee 2 under the title Development of molecular genetic classification of breast cancer, and obtained ethics approval by the Northwest—Greater Manchester Central Research Ethics Committee under the title; Nottingham Health Science Biobank, reference number 15/NW/0685.

## Author Contributions

AI stained and scored all the cases, took the lead in writing the article, data analysis and interpretation, SM helped with double scoring, ER: conceived and planned the study, contributed to data interpretation, made critical revisions, and approved the final version. All authors contributed to writing the article and approved the final version.

## Conflict of Interest

The authors declare no conflicts of interest.

## Data Availability

The authors confirm the data that has been used in this work and is available on reasonable request.
